# Endoglin is not a mediator in 7-ketocholesterol-induced endothelial activation in liver sinusoidal endothelial cells in vitro

**DOI:** 10.1007/s00011-026-02307-5

**Published:** 2026-06-22

**Authors:** Petra Fikrova, Katarina Tripska, Samira Eissazadeh, Martina Vasinova, Adela Diepoltova, Jana Urbankova Rathouska, Ivana Nemeckova, Radim Havelek, SeyedehNiloufar Mohammadi, Ivone Cristina Igreja Sa, Petr Nachtigal

**Affiliations:** 1 Department of Biological and Medical Sciences, Faculty of Pharmacy in Hradec Kralove, Charles University, Heyrovskeho 1203, 500 05 Hradec Kralove, Czech Republic; 2 Department of Biochemistry, Faculty of Medicine in Hradec Kralove, Charles University, Hradec Kralove, Czech Republic; 3 Department of Clinical Microbiology, Faculty of Medicine in Hradec Kralove, Charles University, Hradec Kralove, Czech Republic

**Keywords:** Endoglin, ICAM-1, 7-Ketocholesterol, Liver sinusoidal endothelial cells, Cell adhesion

## Abstract

**Background:**

Endoglin (ENG) is expressed in liver sinusoidal endothelial cells (LSECs), where it regulates endothelial activation and inflammation. The 7-ketocholesterol (7-K), a cholesterol oxidation product, accumulates in the liver and induces endothelial dysfunction. ENG has been shown to play an important role in adhesion processes in other endothelial models but its role in LSECs upon 7-K exposure remains unclear. We hypothesized that 7-K treatment of LSECs would confirm the critical role of ENG in endothelial activation, potentially outweighing the contributions of the cell adhesion molecules VCAM-1 and ICAM-1.

**Methods:**

Human LSECs were exposed to 25 µM 7-K. Protein expression and monocyte adhesion were assessed by flow cytometry, and soluble ENG was measured by ELISA.

**Results:**

7-K reduced ENG expression, while ICAM-1 was markedly upregulated. 7-K increased monocyte adhesion to LSECs, which was abrogated by ICAM-1, but not by ENG neutralization.

**Conclusion:**

We showed that 7-K promotes a pro-inflammatory phenotype defined by ICAM-1, rather than ENG. Thus, we propose that individual roles of ENG, ICAM-1, and VCAM-1 must be carefully considered when studying endothelial dysfunction.

## Introduction

Endoglin is a co-receptor for transforming growth factor-beta (TGF-β). There are two distinct forms: membrane endoglin (ENG) and soluble endoglin (sENG). Previous studies showed a critical role of ENG expression, regulation, and pharmacological modulation in endothelial dysfunction, adhesion, and transmigration in endothelial cells after various stimuli [1]. Moreover, alteration and modulation of ENG expression in LSECs were related to the endothelial cell activation and inflammation in the liver [2, 3]. Moreover, sENG cleaved by matrix metalloproteinase-14 (MMP-14) [4], has been identified as a potential biomarker of endothelial dysfunction [5], and liver diseases such as metabolic dysfunction-associated steatohepatitis (MASH) [6]. However, the role of ENG in mediating hepatic endothelial activation in LSECs remains unexplored.

LSECs are highly specialized endothelial cells lining the hepatic sinusoids, characterized by fenestrations, the absence of a basement membrane, and low expression of adhesion molecules, which mediate exchange with hepatocytes and regulate hepatic immunity, among other roles [7]. During liver diseases, such as MASH or fibrosis, the liver microenvironment becomes enriched with oxidized particles, including oxysterols, such as 7-ketocholesterol (7-K) [8]. 7-K is a major oxidation product of cholesterol, formed during the oxidative modification of low-density lipoproteins (oxLDL). It exerts cytotoxic and pro-inflammatory effects, particularly in vascular endothelial cells [9].

Endothelial activation is also marked by increased expression of adhesion molecules, specifically vascular cell adhesion molecule 1 (VCAM-1) and intercellular cell adhesion molecule 1 (ICAM-1), which are crucial for leukocyte adhesion and transmigration during inflammation [10] and has been associated with liver inflammation and fibrosis progression [11].

We hypothesized that 7-K treatment of LSECs would confirm the critical role of ENG in endothelial activation, potentially outweighing the contributions of VCAM-1 and ICAM-1.

## Methods

Human hepatic sinusoidal endothelial cells (LSECs) from Innoprot were used. Cholesterol-induced endothelial dysfunction was simulated by treatment with 25 µM 7-ketocholesterol (7-K, Merck) for 16 h, followed by protein and functional assays.

Surface expression of ENG, VCAM-1, ICAM-1, and MMP-14 was analyzed by flow cytometry using fluorophore-conjugated antibodies against human ENG, VCAM-1, ICAM-1 (all BioLegend), and MMP-14 (R&D Systems).

Leukocyte adhesion was assessed using fluorescently labeled THP-1 cells (ECACC), which were co-incubated with LSEC monolayers for 1 h. Neutralizing antibodies against ICAM-1, VCAM-1 (both eBioscience), and ENG (TRC105, TRACON Pharmaceuticals), with corresponding isotype controls were used.

Soluble endoglin (sENG) concentration in supernatants was determined by ELISA (R&D Systems).

## Results

**Fig. 1:**
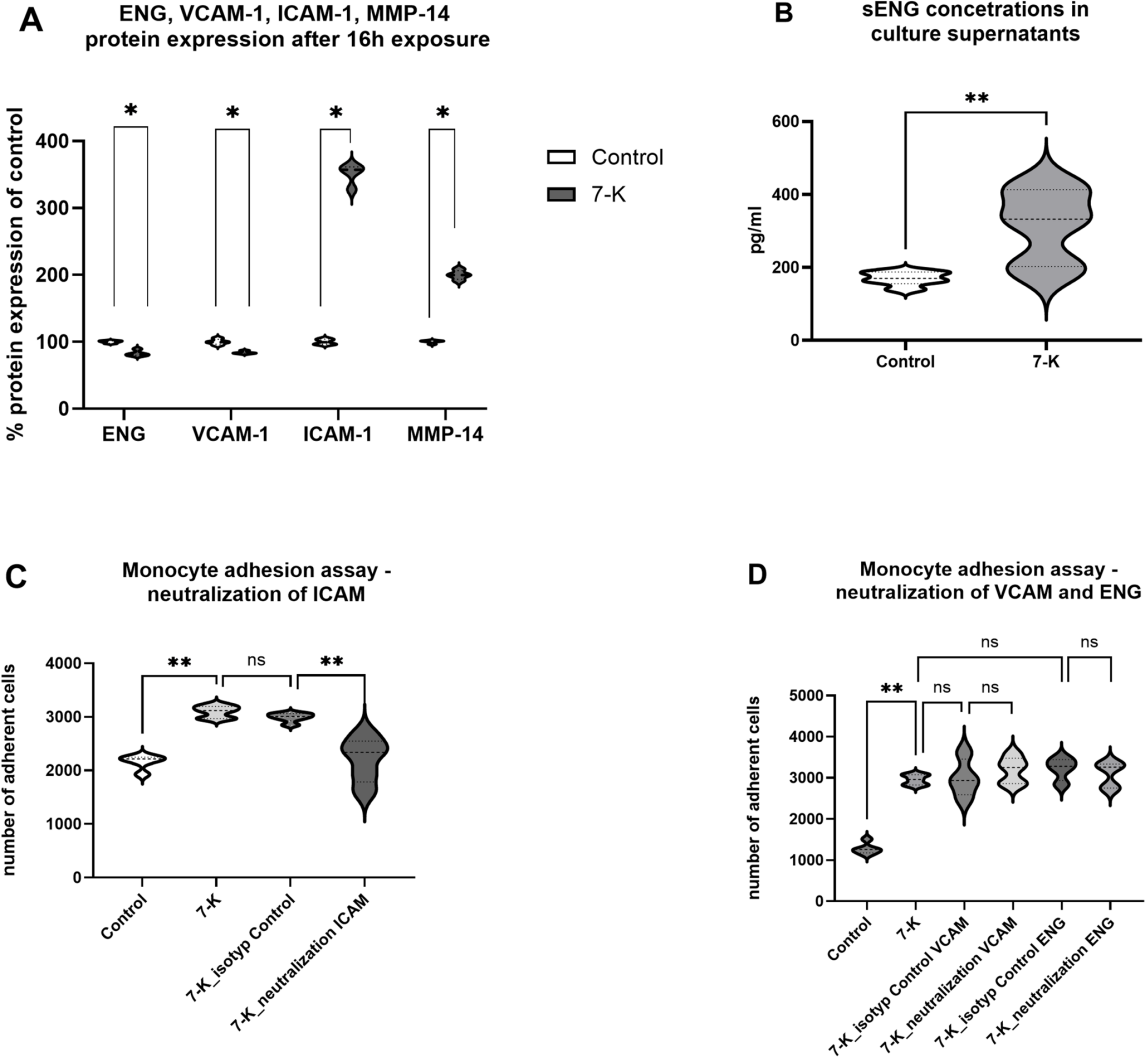
Membrane ENG, VCAM-1, ICAM- 1, and MMP-14 protein expression assessed by flow cytometry **A** sENG concentrations in supernatants measured by ELISA **B** Adhesion of THP-1 cells to LSECs treated with 7-K with or without ICAM-1 blocking antibody (**C**), VCAM-1 blocking antibody, or ENG blocking antibody (**D**). Data are presented as median with interquartile range (n = 4–6); representative of three independent experiments is shown. Mann–Whitney test: ns, *p* > 0.05; *p ≤ 0.05; **p ≤ 0.01

Membrane ENG and VCAM-1 protein expression was significantly decreased, whereas ICAM-1 and MMP-14 expression was significantly increased after 16 h of 7-K treatment compared to control (Fig. 1A). sENG concentration was significantly increased in 7-K-treated cells compared to control (Fig. 1B).

7‑K treatment of LSECs significantly increased monocyte adhesion compared to control and neutralization of ICAM‑1 markedly reduced 7‑K‑induced monocyte adhesion, identifying ICAM‑1 as the principal mediator of this effect (Fig. 1C). In contrast, neutralization of VCAM‑1 or ENG did not significantly affect monocyte adhesion following 7‑K treatment (Fig. 1D).

## Discussion

We provide the first evidence that ICAM-1, but not ENG or VCAM-1, plays a critical role in mediating monocyte adhesion to LSECs in association with endothelial activation following 7-K treatment.

Previous studies have showed that 7-K increased ENG expression and induced inflammation in human aortic endothelial cells, followed by increased adhesion and transendothelial migration of monocytes, which was effectively abrogated by ENG inhibition [12] or neutralization with anti-ENG antibody TRC105, regardless of increased expression of cell adhesion molecules [13].

Here, we demonstrated a significant increase in sENG concentration in supernatants, accompanied by a reduction in membrane ENG protein expression, which indicates enhanced cleavage of ENG by increased MMP-14 [14]. Interestingly, a previous study in LSECs treated with oxLDL, which contains 7-K as a major component, reported increased ENG protein expression [2]. This discrepancy may reflect the complexity of oxLDL particles, which contain multiple bioactive lipids and modulate broader signaling pathways [9] compared to treatment with 7-K alone.

Despite decreased ENG expression after 7-K treatment, we observed increased monocyte adhesion to LSECs, suggesting that ENG was not responsible for this effect under these conditions, which was also supported by the adhesion assay with ENG-neutralizing antibody. Thus, we shifted our focus to the role of VCAM-1 and ICAM-1.

We showed that 7-K treatment significantly upregulated ICAM-1 expression while suppressing VCAM-1 expression in LSECs. Importantly, the increased expression of ICAM-1 was found to be functionally relevant, as it translated into enhanced monocyte adhesion, which was effectively reversed by ICAM-1 neutralization, whereas VCAM-1 neutralization had no effect. These findings highlight the central role of ICAM-1 in mediating 7-K–induced leukocyte recruitment to the hepatic endothelium. This is supported by a well-documented role of ICAM in leukocyte adhesion and transmigration, and its overexpression has been linked to liver inflammation and fibrogenesis in both research and clinical practice [15].

Overall, we demonstrate here for the first time that 7-K promotes a pro-inflammatory phenotype in LSECs, where monocyte adhesion is mediated primarily through ICAM-1 rather than ENG or VCAM-1. These findings highlight the distinct roles of ENG, VCAM-1 and ICAM-1, emphasizing that their contributions to endothelial activation, inflammation, and leukocyte adhesion may vary significantly depending on the pathological context. This study paves the way for targeted interventions to control hepatic endothelial inflammation and activation.

## Data Availability

The original contributions of this study are detailed in the article; further inquiries can be directed to the corresponding author.
